# Impact of cachexia on oncologic outcomes of sarcopenic patients with upper tract urothelial carcinoma after radical nephroureterectomy

**DOI:** 10.1371/journal.pone.0250033

**Published:** 2021-04-21

**Authors:** Hao-Wei Chen, Yu-Chen Chen, Li-Hwa Yang, Ming-Chen Paul Shih, Ching-Chia Li, Kuang-Shun Chueh, Wen-Jeng Wu, Yung-Shun Juan

**Affiliations:** 1 Graduate Institute of Clinical Medicine, College of Medicine, Kaohsiung Medical University, Kaohsiung, Taiwan; 2 Department of Urology, Kaohsiung Medical University Hospital, Kaohsiung Medical University, Kaohsiung, Taiwan; 3 Department of Radiology, Kaohsiung Medical University Hospital, Kaohsiung Medical University, Kaohsiung, Taiwan; 4 Department of Urology, Kaohsiung Municipal Ta-Tung Hospital, Kaohsiung, Taiwan; Chang Gung Memorial Hospital and Chang Gung University, Taoyuan, Taiwan, TAIWAN

## Abstract

**Objectives:**

To investigate the prognostic significance of sarcopenic cachexia compared to sarcopenia without cachexia in the outcomes of upper urinary tract urothelial carcinoma (UTUC) patients treated with radical nephroureterectomy (RNU).

**Materials and methods:**

Between 2011 and 2016, 163 patients with UTUC who received RNU at a tertiary medical center were included. Pre-operatively clinical data, history, and abdominal computer tomography scans were analyzed retrospectively. The diagnosis of sarcopenia was based on abdominal computed tomography data on the patient’s skeletal muscles. Outcomes of relapse-free, cancer-specific, and overall survival were analyzed by multivariate Cox regression.

**Results:**

After adjusting for age, sex, pre-operatively estimated glomerular filtration rate, body mass index, underlying diseases, tumor grade, and tumor stage, cachexia was a significant poor prognostic factor for relapse-free survival (hazard ratio [HR]: 18.5, 95% confidence interval [CI]: 2.87–118, *p* = 0.002) and cancer-specific survival (HR: 26.6, 95% CI: 4.04–175, *p* = 0.001). In contrast, sarcopenia without cachexia was not a significant predictor of cancer outcomes.

**Conclusions:**

To date, this is the first study to investigate the effect of cachexia among sarcopenic patients with UTUC treated with RNU. We identified the prognostic significance of cachexia on outcomes. Indeed, when UTUC is treated with RNU, we should evaluate not only sarcopenia status but also cachexia. The low survival rate among patients with UTUC complicated with cachexia deserves attention.

## Introduction

Although radical nephroureterectomy (RNU) is the standard of care for patients with non-metastatic upper urinary tract urothelial carcinoma (UTUC), the prognosis after RNU is relatively poor. Thus, identifying high-risk patients as candidates for adjunctive therapies is important to improve oncological outcomes. Host factors play an important role, but tumor factors including the pT and pN stages, tumor grade, and lymphovascular invasion are essential [1–3].

Patients with cancer were reported to be at an increased risk for muscle loss via two distinct mechanisms: (1) cytokine-mediated degradation of muscle and adipose depots, called cachexia, which affects 25–80% of patients with cancer and is one of the main cause of cancer death in about 30–50% of the patients and (2) age-associated decrease in muscle mass related to changes in muscle synthesis signaling pathways, called sarcopenia, with a prevalence of 15–50% among patients with cancer [4–6].

Recent studies revealed a novel concept that sarcopenia was associated with unfavorable prognosis in patients with various cancers of the respiratory, gastrointestinal, and urinary tracts [7–13]. However, these studies focused on the comparison of patients with or without sarcopenia. An important factor related to cancer and muscle loss, i.e., cachexia, has not been adequately assessed. To date, no study has reported the effect of cachexia on oncological outcomes in cancer patients with sarcopenia. Therefore, this study investigated the prognostic significance of sarcopenia complicated with cachexia compared to sarcopenia without cachexia in the outcomes of patients with operable UTUC treated with RNU.

## Materials and methods

### Study population

Between January 2011 and December 2016, patients with non-distant metastatic UTUC diagnosed by pre-operative abdominal computer tomography (CT) and surgical pathology specimen, treated with RNU in a tertiary medical center were included. Patients who received neoadjuvant chemotherapy and patients with end-stage kidney disease before undergoing RNU were excluded. Patients underwent standard RNU either by open or laparoscopic procedure with bladder cuff excision. The lymph nodes were dissected if they were enlarged on preoperative evaluation or intraoperative inspection. The ethics committee waived the need for informed consent as stated by the federal regulation of Department of Health and Human Services (code title 45, 46.116) and this study was approved by the Institutional Review Board at Kaohsiung Medical University Hospital (ID: KMUHIRB-E(I)-20170240).

### Evaluation: Definition of cachexia and sarcopenia

Pre-operative clinical data, history, and abdominal CT data were retrospectively recorded and analyzed. Based on the objective definition proposed by the Cachexia Consensus Conference [14], cachexia was defined as weight loss of at least 5% in ≤12 months or low body mass index (BMI) ranging from 18.5 to 22 kg/m^2^ with the presence of any three of the following: fatigue, anorexia, decreased muscle strength, lab evidence of anemia (Hb <12 g/dL), hypoalbuminemia (<3.2 g/dL), or elevated levels of C-reactive protein (CRP).

**Fig 1:**
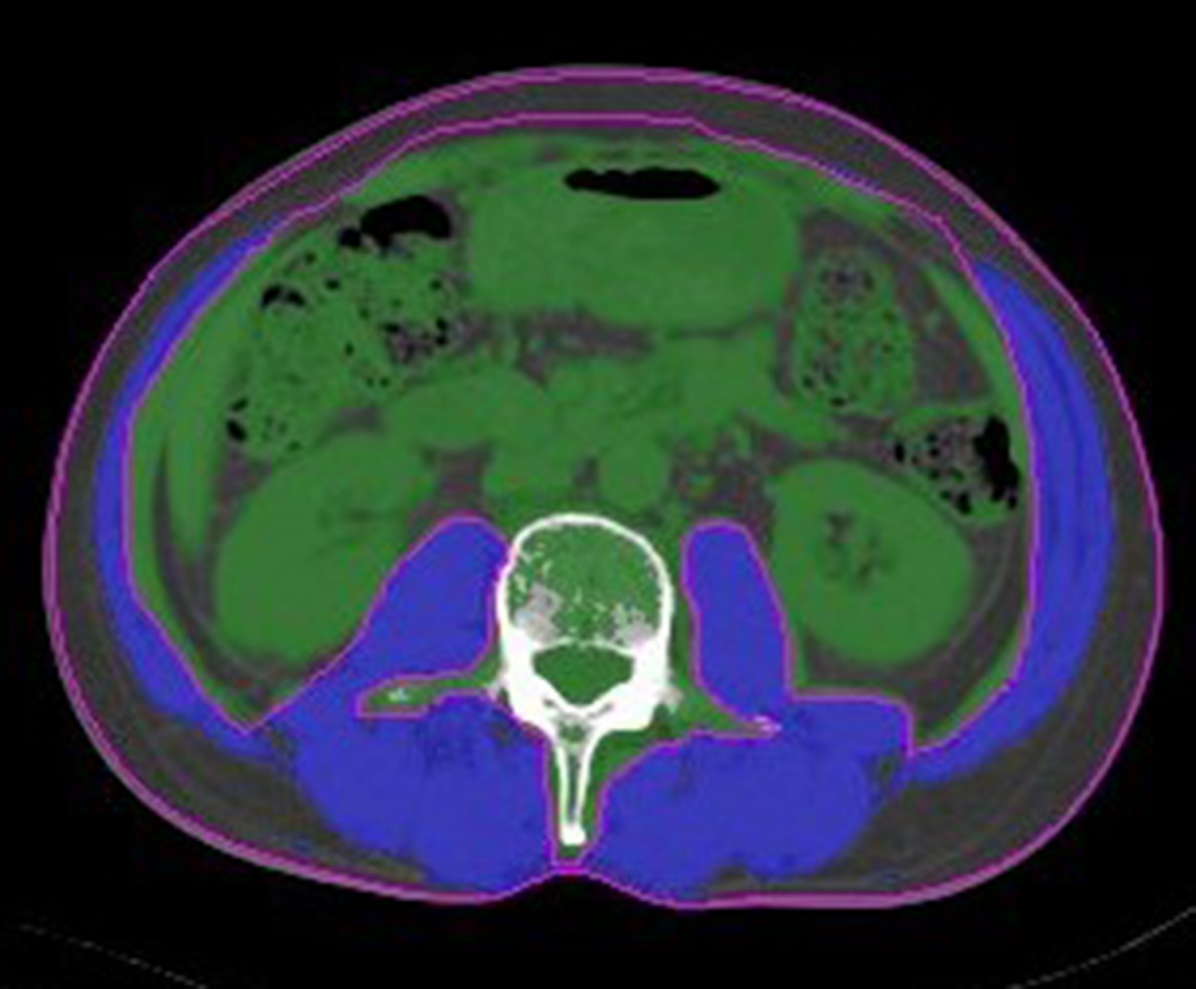
Two adjacent axial CT images of 5-mm slice thickness at the third lumbar vertebrae were used to analyze total muscle cross-sectional area (cm^2^) for each patient. The total muscle cross-sectional area (blue area in the figure) included the abdominal oblique, rectus abdominus, quadratus lumborum, psoas, and erector spine.

Skeletal muscle index (SMI) (cm^2^/m^2^), calculated by normalizing skeletal muscle area for height in meters squared, was used to evaluate sarcopenia alongside abdominal CT data, in our study (Fig 1). We used two adjacent axial CT images with 5-mm slice thickness at the third lumbar vertebrae before RNU to analyze the total muscle cross-sectional area (cm^2^). The total muscle cross-sectional area included the abdominal oblique, rectus abdominus, quadratus lumborum, psoas, and erector spinae. According to Martin et al. [15], sarcopenia was defined as SMI of <43 cm^2^/m^2^ for men with body mass index (BMI) <25 kg/m^2^, SMI <53 cm^2^/m^2^ for men with BMI ≥25 kg/m^2^, and SMI <41 cm^2^/m^2^ for women.

### Follow-up and outcome assessment

The patients underwent urine cytology and cystoscopy every 3 months for 2 years after RNU, every 6 months for the next 3 years, and every 6–12 months thereafter. CT, magnetic resonance imaging (MRI), and/or excretory urography were also performed every 6 months for 5 years and annually thereafter. The outcomes were relapse-free survival (RFS), cancer-specific survival (CSS), and overall survival (OS). RFS was defined as the time from RNU to the first instance of local recurrence, metastasis, or death due to any cause. CSS and OS were defined as the time from the RNU to death due to cancer-related causes or any cause, respectively.

### Statistical analysis

We analyzed the relationship between clinicopathological parameters including body condition (sarcopenia with cachexia, sarcopenia without cachexia, and no sarcopenia), sex, comorbidity (diabetes mellitus, hypertension, heart disease, lung disease), and tumor stage using McNemar’s test. The continuous variables of BMI, estimated glomerular filtration rate (eGFR), and age were analyzed by Mann-Whitney *U* test. Outcomes of RFS, CSS, and OS were analyzed using the Cox regression model. Risk was expressed as hazard ratio (HR) with 95% confidence interval (CI). All analyses were performed using the SPSS version 22 software, and differences with a p value of <0.05 were considered statistically significant.

## Results

The patients’ characteristics are summarized in Table 1. Among the 163 included patients, 132 patients were sarcopenic (80%). The sarcopenic patients were older (mean age: 69.6 vs. 63.7 years, p = 0.003), were more likely to be female (58 vs. 33%, p = 0.016), had lower BMIs (23.4 vs. 25.5%, p = 0.001), and had worse renal function (mean eGFR: 52.9 vs. 65.5, p = 0.016) than the non-sarcopenic patients. All non-sacropenic patients did not have cachexia. Further, sarcopenic patients with cachexia were younger (mean age: 67.5 vs. 70.7 years, p = 0.034), had lower BMIs (20% vs. 25.1%, p < 0.001), and worse renal function (mean eGFR: 46.1 vs. 56.1, p = 0.029) than the sarcopenic patients without cachexia (Table 2). During the follow-up (median 30 months, range 1–86), 51 patients died of other causes and 29 died of UTUC.

**Table 1:** Characteristics of the patients.

	All patients (N = 163)		
Characteristics	With Sarcopenia (n = 132)	Without sarcopenia (n = 31)	*P*-value
Mean age, years (range)	69.6 (42–90)	63.7 (46–87)	0.003
Mean BMI, kg/m^2^ (range)	23.4 (13.8–39.4)	26.5 (20.4–39.5)	0.001
Sex, n (%)			0.016
Male	56 (42)	21 (67)	
Female	107 (58)	142 (33)	
eGFR, mL/min/1.73 m^2^ (range)	52.9 (8.4–159)	65.5 (15–136)	0.016
Comorbidity			
DM, n (%)	32 (24)	9 (29)	0.6
Hypertension, n (%)	66 (50)	18 (58)	0.4
Heart disease, n (%)	22 (17)	5 (16)	1
Lung disease, n (%)	6 (4)	1 (3)	1
Pathologic tumor grade			0.5
Low grade, n (%)	26 (20)	8 (26)	
High grade, n (%)	106 (80)	23 (74)	
Pathologic tumor stage			0.2
Local, n (%)	89 (67)	25 (80)	
Locally advanced, n (%)	43 (33)	6 (20)	

Abbreviations: BMI = body mass index; eGFR = estimated glomerular filtration rate; DM = diabetes mellitus.

**Table 2:** Characteristics of sarcopenic patients.

	Sarcopenia (n = 132)		
Characteristics	With cachexia (n = 45)	Without cachexia (n = 87)	*P*-value
Mean age, year (range)	67.5 (44–84)	70.7 (42–90)	0.034
Mean BMI, kg/m^2^ (range)	20 (13.8–26.3)	25.1 (19.5–39.5)	<0.001
Sex, n (%)			0.6
Male	21 (46)	35 (40)	
Female	24 (54)	52 (60)	
eGFR, mL/min/1.73 m^2^ (range)	46.1 (14–104)	56.1 (8.4–159)	0.029
Comorbidity			
DM, n (%)	11 (24)	21 (24)	1
Hypertension, n (%)	19 (42)	47 (54)	0.3
Heart disease, n (%)	6 (13)	16 (18)	0.6
Lung disease, n (%)	3 (6)	3 (3)	0.4
Pathologic tumor grade			0.1
Low grade, n (%)	5 (11)	21 (24)	
High grade, n (%)	40 (89)	66 (76)	
Pathologic tumor stage			0.018
Local, n (%)	24 (53)	65 (75)	
Locally advanced, n (%)	21 (47)	22 (25)	

Abbreviations: BMI = body mass index; eGFR = estimated glomerular filtration rate; DM = diabetes mellitus.

In the patients without sarcopenia, there were 23 patients had high grade tumor (74%) and 6 patients had had locally advanced tumor stage (20%), respectively (Table 1). In the follow-up of patients without sarcopenia, seven patients died of other causes and two died of UTUC. In the sarcopenic patients without cachexia, there were 66 patients had high grade tumor (76%) and 22 patients had had locally advanced tumor stage (25%), respectively (Table 2). For sarcopenic patients without cachexia during the follow-up, there were 9 patients died of other causes and 2 died of UTUC. In the sarcopenic patients with cachexia, there were 40 patients had high grade tumor (89%) and 21 patients had had locally advanced tumor stage (47%), respectively (Table 2). Lastly, in the follow-up of sarcopenic patients with cachexia, six patients died of other causes and 25 died of UTUC.

**Fig 2:**
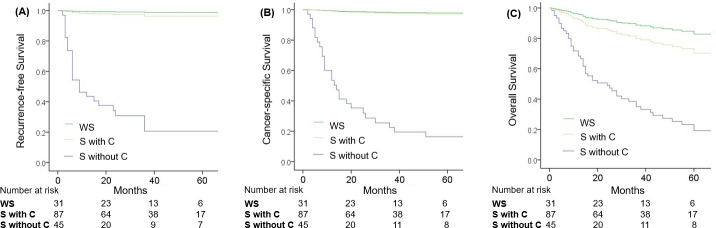
Kaplan-Meier curves for RFS, CSS, and OS of UTUC patients after RNU. Abbreviations: WS = without sarcopenia, S with C = sarcopenia with cachexia, S without C = sarcopenia without cachexia.

Results of multivariate Cox regression analysis (Table 3) showed that sarcopenia with cachexia was a significantly independent prognostic factor for both RFS (hazard ratio [HR]: 18.5, 95% confidence interval [CI]: 2.87–118, p = 0.002) and CSS (HR: 26.6, 95% CI: 4.04–175, p = 0.001), but not for overall survival (HR: 2.68, 95% CI: 0.93–7.67, p = 0.068). However, sarcopenia without cachexia was not a significant predictor for RFS (HR: 0.21, 95% CI: 0.02–2.42, p = 0.2), CSS (HR: 0.46, 95% CI: 0.06–3.58, p = 0.05), and OS (HR: 0.41, 95% CI: 0.15–1.04, p = 0.06). In addition, tumor grade was significantly associated with RFS, CSS, and OS; BMI and sex were significantly associated with CSS; and comorbidity with heart disease were significantly associated with OS. Fig 2A–2C showed the Kaplan-Meier survival curves of RFS, CSS, and OS according to the condition of sarcopenia and cachexia in the UTUC patients.

**Table 3:** Multivariate Cox regression model of RFS, CSS, and OS of UTUC patients after RNU.

	RFS		CSS		OS	
	HR (95% CI)	*P*-value	HR (95% CI)	*P*-value	HR (95% CI)	*P*-value
**Status**						
Without sarcopenia	Ref.		Ref.		Ref.	
Sarcopenia with cachexia	18.5 (2.8 7–118)	0.002	26.6 (4.04–175)	0.001	2.68 (0.93–7.67)	0.068
Sarcopenia without cachexia	0.21(0.02–2.42)	0.2	0.46 (0.06–3.58)	0.5	0.41 (0.15–1.04)	0.06
**Age**	0.99 (0.95–1.05)	0.9	0.99 (0.94–1.05)	0.8	1.01 (0.97–1.04)	0.8
**BMI**	1.11 (0.97–1.27)	0.12	1.17 (1.02–1.34)	0.027	1.01 (0.91–1.12)	0.9
**Sex**		0.2		0.005		0.06
Female	Ref.		Ref.		Ref.	
Male	2.05 (0.9–4.65)		3.39 (1.44–7.96)		1.8 (0.97–3.35)	
**eGFR**	0.99 (0.97–1.02)	0.5	0.99 (0.96–1.01)	0.2	0.99 (0.98–1.01)	0.4
**Comorbidity**						
Diabetes mellitus	1.21 (0.45–3.24)	0.7	0.96 (0.36–2.57)	0.9	1.33 (0.7–2.56)	0.4
Hypertension	0.62 (0.27–1.44)	0.3	0.78 (0.34–1.76)	0.5	0.92 (0.51–1.65)	0.8
Heart disease	1.79 (0.64–5.05)	0.3	1.89 (0.67–5.37)	0.2	2.53 (1.18–5.42)	0.017
Lung disease	0.41 (0.05–3.32)	0.4	0.42 (0.05–3.43)	0.4	0.53 (0.12–2.28)	0.4
**Tumor stage**		0.014		0.004		0.002
Localized	Ref.		Ref.		Ref.	
Locally advanced	3.19 (1.27–7.99)		3.79 (1.52–9.48)		2.68 (1.43–4.99)	

Abbreviations: RFS = relapse-free survival; CSS = cancer-specific survival; OS = overall survival; UTUC = upper tract urothelial carcinoma; RNU = radical nephroureterectomy; HR = Hazard ratio; CI = confidence interval; eGFR = estimated glomerular filtration rate.

## Discussion

Although sarcopenia is related with unfavorable prognosis in the case of some cancers [13,16–18], no study has analyzed cachexia that is frequently combined with sarcopenia, resulting in confounding bias. To our knowledge, this is the first study to investigate this prognostic factor among sarcopenic UTUC patients treated with RNU. We found that cachexia significantly affects the outcomes of patients with cancer–the prognostic significance of sarcopenia with cachexia was evident in the RFS and CSS.

Sarcopenia, which was reported to be associated with decreased aerobic capacity, metabolic rate, and strength, is the age-related loss of muscle tissue [5,19]. In general, sarcopenia occurs with aging, where muscle loss begins slowly around 30 years old and accelerates after 65 years old [5,20,21]. Sarcopenia is particularly relevant for patients with cancer as the incidence of disease increases with age [5,22]. Increased inflammation and poor nutrient intake related with cancer may exacerbate muscle loss among older individuals [5]. In a similar vein, in this retrospective study, patients with sarcopenia were significantly older than those without sarcopenia in the UTUC patient group treated with RNU.

Many studies have reported the prognostic significance of sarcopenia for UTUC patients who underwent RNU [1–3] by comparing the outcomes of sarcopenic and non-sarcopenic patients. However, these studies did not evaluate another important condition that frequently occurs in cancer patients–cachexia.

Cachexia, a muscle loss mechanism distinct from sarcopenia, is a multifactorial syndrome defined by loss of skeletal muscle mass with or without fat wasting that cannot be reversed by nutrition support in the context of chronic systemic inflammation and metabolic alterations, affecting approximately 50% of patients with cancer [6]. Cachexia may be present at cancer diagnosis, but it worsens as the cancer progresses or during cancer treatment [5]. While cachexia and sarcopenia have different etiologies, they also have different treatment options [5], making it important to accurately identify each condition. In the present study, we identified cachexia among sarcopenic patients and found that sarcopenia complicated with cachexia was significantly associated with poor RFS and CSS.

Few studies have demonstrated the prognostic roles of CRP level as one of the cachexia-related criteria for UTUC patients treated with RNU [3,23,24].These studies analyzed only the CRP level instead of using all cachexia-related criteria, which led to controversial results: some studies identified CRP level as a useful biomarker of poor prognosis in UTUC patients treated with RNU [23,24], while others reported the opposite [3]. In the present study, we retrospectively reviewed the included patients and identified those with cachexia using the objective definition proposed by the Cachexia Consensus Conference [14].

In clinical practice, despite the differing etiologies of cachexia and sarcopenia, each state is potentially exacerbated by the other during cancer treatment. In cancer patients, for example, sarcopenia may lead to cachexia, resulting in the depletion of already low muscle mass. Alternatively, a patient with cachexia may also lose muscle mass with increasing age. Hence, we suggest that UTUC patients preoperatively diagnosed with sarcopenia should be further assessed to check for the presence of cachexia. Identifying cachexia may be helpful for counseling patients on their personalized risks and in determining therapeutic strategies. Disease recurrence and adjuvant chemotherapy should be considered among sarcopenic patients with cachexia.

Various definitions of sarcopenia were used based on the findings of CT images among published studies [15,25], resulting in heterogeneity and limitations when addressing the clinical impact of sarcopenia on cancer patients. Recently, it is believed that BMI-incorporated sarcopenia, defined by Martin et al. [15] may be applicable to populations with differing BMIs and fit contemporary clinical settings, in which the prevalence of obesity is increasing [16]. However, the limitation of our study is that the SMI cutoffs in Martin et al.’s study involving a Western population may be inappropriate for Asians because Asians have lower SMIs than Westerners [8,26]. Recently, the dual X-ray absorptiometry, handgrip strength (<26 kg for men and <18 kg for women), and usual gait speed (<0.8 m/s) were used to assess sarcopenia in Consensus Report of the Asian Working Group for Sarcopenia after 2014 [27]. Although we believe these can evaluate the Asian patients more accurately, these 3 tests were not routine examination in clinical practice. Further studies are needed to establish optimal sarcopenia cutoffs for Taiwanese populations using CT images.

The other limitations of the present study were its retrospective design and the small patient population. Recently, the scoring systems cachexia score (CASCO) became available for use after 2011 [28], the additional physical performance (PHP) and quality of life (QoL) were criteria that could have been useful in the assessment of the severity of cachexia. However, multiple questionnaires were needed to assess physical performance (PHP) and quality of life (QoL). Due to retrospective design, we could not assess the PHP and QoL of the patients. Besides, all of our patient had no adjuvant therapy after radical nephroureterectomy. So, we didn’t assess the adjuvant therapy influence the outcome and different treatment options of cachexia and sarcopenia. Further studies prospective studies are needed to validate the predictive value of cachexia among sarcopenic patients with UCUT who are undergoing RNU. The current preliminary results need to be validated by large multicenter studies.

## Conclusion

Cachexia was a prognostic indicator of poor RFS and CSS among UTUC patients who underwent RNU surgery. Indeed, in UTUC patients treated with RNU, we should evaluate not only sarcopenia status but also the status of cachexia. The risk of poor survival in cancer patients with sarcopenia complicated with cachexia should be examined in future studies.
